# Navigating Pregnancy With Uterine Fibroids: A Case Study

**DOI:** 10.7759/cureus.64793

**Published:** 2024-07-18

**Authors:** Dharmesh J Patel, Kamlesh Chaudhari, Neema Acharya, Deepti Shrivastava, Apoorva Dave, Archan Patel

**Affiliations:** 1 Department of Obstetrics and Gynaecology, Jawaharlal Nehru Medical College, Datta Meghe Institute of Higher Education and Research, Wardha, IND

**Keywords:** obstetric ultrasound, reproductive health, obstetric outcomes, pregnancy complications, uterine fibroids

## Abstract

Benign uterine tumors, known as leiomyomas or uterine fibroids, can result in severe pain, bleeding, and infertility. They impact a woman's overall well-being, ability to conceive, and the course of her pregnancy. Fibroids are associated with increasing maternal age. When a patient with fibroids is considering pregnancy, ultrasonography and a detailed pelvic examination should be performed to determine the size and location of any fibroids. This case study details a 30-year-old female patient who had fibroids during her pregnancy and responded well to treatment.

## Introduction

Fibroids, also known as leiomyomas, are the most frequent pelvic tumors during pregnancy, with a prevalence of 10.7% in the first trimester [1,2]. Fibroids can affect a woman's daily activities if she becomes symptomatic during pregnancy and can also impact her fertility and obstetric outcomes. Among women of reproductive age, 35-77% are affected by fibroids. Despite the high prevalence, many fibroids remain asymptomatic during reproductive years. As women age, particularly around 50 years old, the prevalence increases to 70-80%, often accompanied by symptoms [3,4]. In 5-10% of fertile patients, fibroids are present, but only 1-2% of infertility cases are attributable to fibroids [5,6]. Fibroids can cause infertility by obstructing the fallopian tubes and preventing oocyte migration, often due to deformities in the uterine cavity that result in hormonal imbalances, altered endometrial receptivity, and impaired endometrial development [7,8].

Pregnancy-induced fibroids were noted in 0.07-10.7% of all pregnancies. According to a study by De Vivo et al., 66.6% of fibroids increase in size during the third trimester, and 71.4% increase in size during the first and second trimesters [9].

Women over 30 years of age who become pregnant for the first time have a higher chance of developing fibroids during pregnancy compared to multiparous women. While most fibroids do not cause complications during pregnancy, they can sometimes lead to severe pain, discomfort, and bleeding. Studies suggest that after delivery, fibroid size decreases significantly, with one study showing a decrease in fibroid size in 72% of women postpartum. During the puerperal period of six months, fibroid volume can decrease by 50% [10,11]. Here, we present a unique case of a 30-year-old woman who complained of an abdominal lump and pain during the first trimester. An ultrasonography revealed a uterine fibroid during her pregnancy.

## Case presentation

A 30-year-old G2P1 female presented to the Obstetrics and Gynecology Department with the chief complaint of lower abdominal pain for the past six days and a palpable mass. The patient had normal menstrual cycles with mild dysmenorrhea. Upon examination, the patient showed no signs of pedal edema, appeared clinically pale, was afebrile, and exhibited no respiratory distress. Her pulse rate was 90/min, and her blood pressure was 100/60 mmHg. Her respiration rate was 17 cycles per minute. A hard, non-tender lump was palpable in the lower left abdomen. Per speculum examination, the vagina and cervix appeared to be in good health. Compared to the projected gestational age, the uterus was larger. On bimanual examination, the os was 2 cm dilated, 25% effaced, and the cervix was soft.

A single intrauterine pregnancy at 18 weeks was confirmed by ultrasound, showing a well-defined iso-echogenic lesion in the uterus, measuring 10 cm with a cystic area, indicative of a fibroid with cystic degeneration. The endometrium measured 5 mm thick. The patient was recommended to have routine ultrasound follow-ups to track the fibroid's growth and the development of the fetus. She was prescribed acetaminophen for pain relief, with instructions to avoid nonsteroidal anti-inflammatory drugs (NSAIDs) due to their potential adverse effects on the fetus. Standard prenatal care was provided, with additional monitoring for signs of preterm labor and placental abruption.

**Figure 1:**
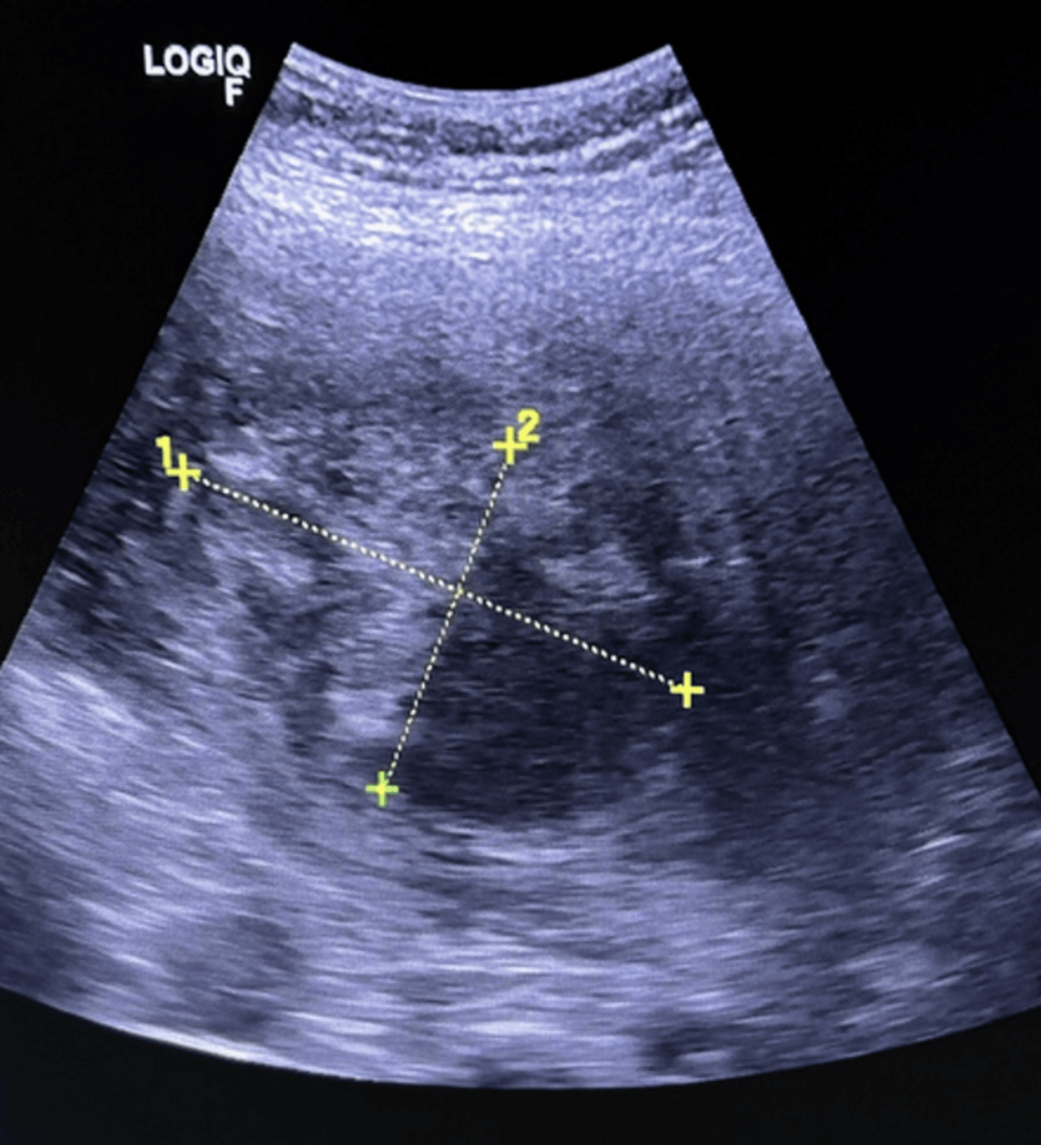
Grey-scale ultrasound image showing a well-defined iso-echogenic lesion in the uterus, measuring 11.5 x 8.8 x 6.3 cm, with a cystic area within, suggestive of a fibroid with cystic degeneration

At 24 weeks' gestation, an ultrasound showed a well-defined iso-echogenic lesion in the uterus, measuring 11.5 x 8.8 x 6.3 cm, with a cystic area, consistent with a fibroid with cystic degeneration (Figure 1).

**Figure 2:**
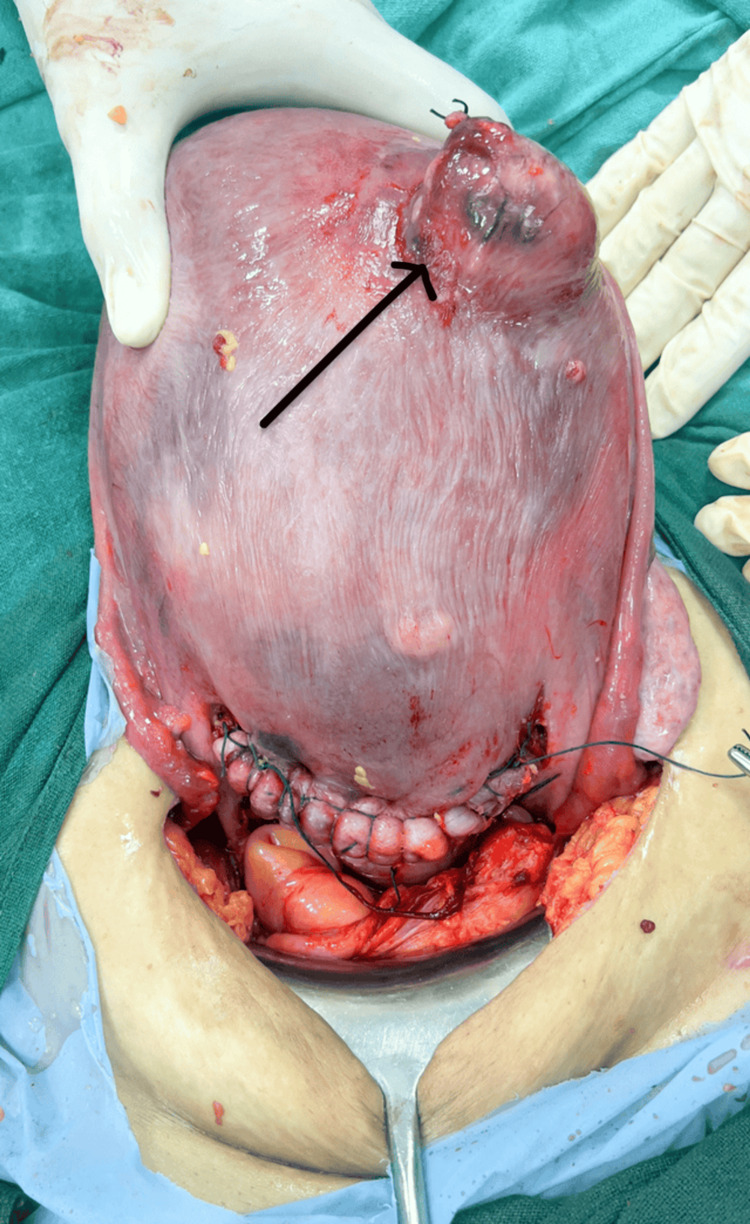
Intramural fibroid (black arrow) noted during lower section cesarean section

The patient experienced intermittent pain and was managed with acetaminophen. During a follow-up at 32 weeks' gestation, the patient reported an increased frequency of pain episodes. The fetus was in cephalic presentation with normal growth parameters. Conservative pain management was continued, and it was planned to deliver the baby by C-section at 38 weeks' gestation to prevent potential complications, such as obstructed labor or emergency cesarean section. The mother and baby had an uneventful recovery. The intramural fibroid was noted during the C-section (Figure 2) but was not removed due to the high risk of hemorrhage. At the six-week postpartum follow-up, the patient was asymptomatic, and the uterus had been involuted appropriately.

## Discussion

The interaction between fibroids and pregnancy is a critical concern for obstetricians due to the potential complications that may arise. This discussion will explore the impact of fibroids on pregnancy, potential complications, management strategies, and outcomes.

Due to the delay in childbearing, which is increasingly common for various reasons, the frequency of uterine fibroids during pregnancy is expected to rise globally [12,13]. Because of poor health-seeking behaviors, social and financial constraints, and anxiety about surgery, women often delay seeking treatment [14,15]. Large or numerous uterine fibroids increase the probability of complications during pregnancy in women who delay seeking treatment [16,17].

Impact of fibroids on pregnancy

Fibroids can influence pregnancy in various ways depending on their number, location, and size within the uterus. They can be classified based on their location as subserosal, intramural, submucosal, and pedunculated.

In most cases, fibroids do not cause any symptoms during pregnancy, meaning they remain asymptomatic and do not interfere with the pregnancy outcome. However, in some instances, they can complicate the pregnancy. Abdominal pain, preterm labor, and postpartum hemorrhage are commonly seen during pregnancy, compared to other signs and symptoms. Other complications include obstructed labor, miscarriages, malposition and malpresentation, and intrauterine growth restriction (Table 1). Fibroids also increase the rate of cesarean sections. Some of these pregnancy complications may necessitate abandoning the standard cautious approach to managing uterine fibroids during pregnancy [18].

**Table 1:** Complications associated with uterine fibroid related to pregnancy

Maternal	Fetal
Pain and discomfort	Miscarriage
Abruptio placenta	Preterm birth
Postpartum hemorrhage	Intrauterine growth restriction (IUGR)
Obstructed labor	Malpresentation
Preterm labor	-
Cesarean delivery	-

In our case, the fibroid enlarged during the third trimester. However, one study found that, in 107 women, fibroid size decreased in the postpartum period, while in 72 women, fibroid size remained the same. There is a higher chance of developing fibroids in the first trimester than in the third trimester.

Cervical fibroids can enlarge during pregnancy and grow towards the uterus. They can cause obstructed labor, preterm labor, antepartum hemorrhage, and sometimes abortion [19-21]. Emergency intervention is required if such situations occur.

Management strategies

The management of fibroids in pregnancy requires a balanced approach to minimize risks to both the mother and the fetus.

Expectant Management

Most cases are managed conservatively with regular monitoring using ultrasound to assess the growth of fibroids and any impact on the pregnancy.

Medication

Analgesics are used for pain management, and in some cases, medications are prescribed to manage symptoms like heavy bleeding.

Surgical Intervention

Myomectomy (surgical removal of fibroids) is generally avoided during pregnancy due to the high risk of complications. However, it may be considered in severe cases with significant symptoms or complications.

Outcomes

The presence of fibroids in pregnancy does not universally predict poor outcomes. Many women with fibroids have uneventful pregnancies and deliver healthy babies. The outcomes largely depend on the size, number, and location of the fibroids, as well as the presence of any associated complications.

Successful Pregnancy

With careful monitoring and appropriate management, many pregnancies proceed to full term with successful outcomes.

Preterm Delivery

Increased surveillance and interventions can help manage preterm labor effectively.

Delivery Method

Vaginal delivery is possible in many cases, but the rate of cesarean sections is higher in women with fibroids due to complications, such as obstructed labor or malpresentation.

In our clinical practice, we frequently encounter patients with large or symptomatic uterine fibroids during pregnancy. We have decided to share our experiences, noting that, while cesarean myomectomy was not necessary for our patient, it remains an option in cases where complications arise.

## Conclusions

We reported a unique case of fibroids during pregnancy that caused symptoms in the patient. The patient received successful care and treatment. Obstetricians and gynecologists must handle uterine fibroids with appropriate attention, taking obstetrical outcomes and pregnancy rates seriously. Individual patient preferences, such as the desire to bear children in the future, should be considered when choosing a course of treatment. Important factors to examine include the number, location, and size of fibroids. The key lies in early detection, regular monitoring, and timely intervention when necessary.
